# IL-6 Baseline Values and Dynamic Changes in Predicting Sepsis Mortality: A Systematic Review and Meta-Analysis

**DOI:** 10.3390/biom15030407

**Published:** 2025-03-13

**Authors:** Norberth-Istvan Varga, Iulia Cristina Bagiu, Dan Dumitru Vulcanescu, Voichita Lazureanu, Mirela Turaiche, Ovidiu Rosca, Adrian Vasile Bota, Florin George Horhat

**Affiliations:** 1 Department of General Medicine, Doctoral School, “Victor Babes” University of Medicine and Pharmacy, Eftimie Murgu Square No. 2, 300041 Timisoara, Romania; norberth.varga@umft.ro; 2 Department of Microbiology, “Victor Babes” University of Medicine and Pharmacy, Eftimie Murgu Square No. 2, 300041 Timisoara, Romania; dan.vulcanescu@umft.ro (D.D.V.); horhat.florin@umft.ro (F.G.H.); 3 Multidisciplinary Research Center on Antimicrobial Resistance (MULTI-REZ), Microbiology Department, “Victor Babes” University of Medicine and Pharmacy, Eftimie Murgu Square No. 2, 300041 Timisoara, Romania; 4 Department XIII, Discipline of Infectious Diseases, “Victor Babes” University of Medicine and Pharmacy, Eftimie Murgu Square No. 2, 300041 Timisoara, Romania; lazureanu.voichita@umft.ro (V.L.); paliu.mirela@umft.ro (M.T.); ovidiu.rosca@umft.ro (O.R.); 5 Methodological and Infectious Diseases Research Center, Department of Infectious Diseases, “Victor Babes” University of Medicine and Pharmacy, Eftimie Murgu Square No. 2, 300041 Timisoara, Romania; 6 Doctoral School, Faculty of Medicine, “Vasile Goldis” Western University, Bulevardul Revolutiei 94, 310025 Arad, Romania; bota.adrian-vasile@student.uvvg.ro

**Keywords:** sepsis, septic shock, sepsis prognosis, cytokine, interleukin-6, IL-6, cytokine storm

## Abstract

Sepsis, a life-threatening condition arising from a dysregulated immune response to infection, is a significant health burden globally. Interleukin-6 (IL-6), an inflammatory cytokine produced by immune cells as a response to infection and tissue damage, plays a key role in the pathogenesis of sepsis. This systematic review and meta-analysis aimed to investigate the association of the baseline plasma levels of IL-6, and the dynamic change in these levels over a timespan of 96 h, with short-term mortality. A systematic literature search was conducted across multiple databases. Studies were included if they assessed the independent prognostic value of IL-6 in adult sepsis patients, used well-defined sepsis criteria, and reported at least one IL-6 measurement. Pooled effect estimates for the association between IL-6 and 28–30-day mortality were determined using logistic regression and AUROC analysis. Thirty-one studies, encompassing 4566 patients, were included. While baseline IL-6 levels and 96 h IL-6 clearance were not significantly associated with mortality risk (pooled OR 1.001, 95% CI 0.999–1.003 and 1.019, 95% CI 0.925–1.112, respectively), AUROC analysis indicated moderate-to-good discriminatory power for both baseline (0.701, 95% CI 0.660–0.742) and 96 h IL-6 clearance (0.828, 95% CI 0.736–0.919) in predicting 28-day mortality. While not a strong independent predictor, IL-6 demonstrates some discriminatory ability, suggesting its potential value in conjunction with other biomarkers.

## 1. Introduction

Sepsis, a life-threatening condition characterized by a dysregulated immune response to infection, represents a major global public health burden [1,2]. With an estimated 49 million cases and 11 million deaths annually, sepsis accounts for nearly 20% of all global deaths [2]. An initial infectious trigger activates a dysregulated immune response, characterized by a potentially fatal cascade of inflammatory mediators [3]. These mediators initiate a complex and dynamic interplay between pro- and anti-inflammatory processes [4]. While the pro-inflammatory response aims to eradicate the invading pathogen, an excessive reaction can lead to collateral tissue damage. A compensatory anti-inflammatory process that also includes various cytokines is set into motion, but it can also be inadequate or exaggerated. This tug-of-war involves not just IL-6, but a chorus of markers, like tumor necrosis factor-alpha (TNF-α), which kickstarts the cytokine storm, and C-reactive protein (CRP), a downstream signal of inflammation, all shaping sepsis’s chaotic course [5]. Sepsis is characterized by the disruption of this delicate balance between pro-inflammatory and anti-inflammatory mechanisms. The systemic inflammatory state that results is marked by widespread endothelial dysfunction, microvascular abnormalities, such as increased permeability, vasodilation, microthrombi formation, and, ultimately, impaired tissue perfusion and organ dysfunction [6,7,8].

Interleukins are a type of cytokine produced by the immune cells (T-helper cells, monocytes and macrophages, B cells, etc.), but also by endothelial and epithelial cells, as a response to infection or injury, and play a central role in the intricate inflammatory network that characterizes sepsis [9]. Their main roles include immune cell development and differentiation, immune cell activation and proliferation, and regulation of the immune response. This includes pro-inflammatory interleukins like IL-1β and IL-8, which amplify the early attack on pathogens, and anti-inflammatory ones like IL-10, which temper the response, alongside IL-6, within a broader family that includes leukemia inhibitory factor (LIF) and oncostatin M (OSM). Interleukin 6 (IL-6) stimulates hepatic synthesis of acute-phase proteins, such as C-reactive protein and fibrinogen [10,11]. It also plays an important role in adaptive immunity by promoting the differentiation of B cells into antibody-secreting plasma cells and influencing the development of various T cell subsects, including pro-inflammatory Th17 cells [10,11,12]. Systemically, IL-6 induces fever and contributes to muscle wasting. In sepsis, IL-6 levels are markedly elevated, due to its increased production by a variety of immune cells: monocytes, macrophages, and endothelial cells [13]. This surge in IL-6 plasma values, coupled with its early appearance in the course of the disease, reflects its utility as a biomarker for sepsis [14].

Building upon the established diagnostic utility of IL-6 in sepsis, the primary focus of this study shifts towards elucidating its prognostic value. Far from acting alone, IL-6 interacts with many other mediators, making its prognostic role trickier to pin down. While elevated IL-6 levels are clearly associated with the presence and severity of sepsis, a more nuanced understanding of their relationship with patient outcomes is needed. Therefore, this systematic review and meta-analysis aims to comprehensively evaluate the available evidence, and to answer pivotal questions regarding the prognostic significance of IL-6 in sepsis. Specifically, it seeks to determine whether higher baseline IL-6 levels are independently associated with an increased risk of mortality in septic patients. Furthermore, it investigates whether the dynamic changes in IL-6 concentrations over time, i.e., IL-6 kinetics, provide additional prognostic information beyond static baseline measurements, as well as determining their ability to predict sepsis mortality. By synthesizing data from original articles reporting serial IL-6 measurements in sepsis, this meta-analysis aims to provide a definitive assessment of IL-6’s role as a prognostic biomarker and its potential to guide clinical decision-making in the management of this life-threatening condition.

## 2. Materials and Methods

### 2.1. Search Strategy

This systematic review and meta-analysis adhered to established best practices. For methodological transparency, the protocol was prospectively registered (PROSPERO ID CRD42024515819), and is reported in accordance with PRISMA guidelines [15]. A comprehensive search strategy across Google Scholar, Cochrane Library, and Pubmed employed the following keywords: “IL-6”, “interleukin-6”, “interleukin-6 in sepsis”, “IL-6 in sepsis”, “sepsis prognosis”, “sepsis prognostic factor”, “sepsis biomarkers”, and “sepsis mortality”. To capture the most relevant advancements, our focus prioritized publications from the past two decades (January 2000–December 2023). The review included original research articles with the following study designs: prospective and retrospective observational cohort studies, cross-sectional studies, and case–control studies.

### 2.2. Study Selection

**Figure 1:**
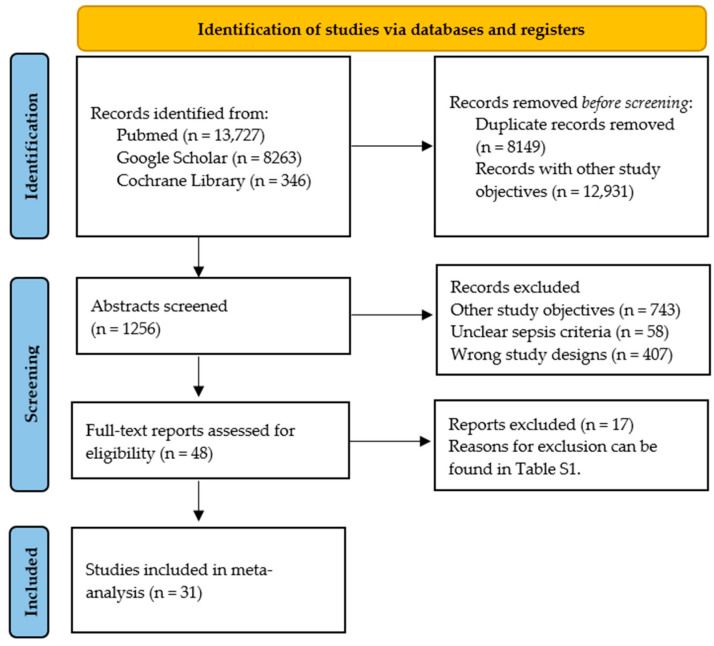
Study selection process according to PRISMA guidelines.

Two independent researchers separately reviewed titles, abstracts, and full-text articles to select papers eligible for our study. If discrepancies arose, a third reviewer resolved those disagreements. Only studies focusing on the prognostic factor’s independent ability to predict patient outcomes were chosen, i.e., studies that measured baseline IL-6 values and/or IL-6 kinetics. The inclusion criteria were as follows: (1) studies that explicitly measured the biomarker’s independent predictive power related to a well-defined outcome (in-hospital mortality or 28–30-day mortality). Only phase 2 confirmatory studies were included (phase 2 studies explore “the independence of the association between a prognostic factor and the outcome of interest … while controlling for confounders” [16]); (2) studies with more than 10 patients and five outcome events; (3) studies only on adult patients (18 years or older); (4) studies that used well-defined criteria for sepsis diagnosis; and (5) studies that reported at least one measurement of the prognostic factor (baseline values and/or kinetics of IL-6 levels). The study selection process, following the PRISMA guidelines, is illustrated in Figure 1.

The exclusion criteria were as follows: studies primarily reporting associations without strong statistical analysis or without controlling for confounders (phase 1 studies), case series, studies unrelated to our primary focus, and studies on patients suffering from immunocompromised conditions.

### 2.3. Data Analysis

Two separate reviewers independently extracted the relevant data from the included studies, and systematically organized them into a table, ensuring a methodical approach for our analysis. The following data were extracted: study ID (first author and year of publication), country, clinical setting, number of participants, outcome events, mean or median age of participants, sex (percentage of males or females), criteria used for sepsis definition, sepsis origin, clinical scores (SOFA, APACHE-II), plasma IL-6 values, logistic regression analysis, area under the receiver operating characteristics (AUROC) curves to determine outcome, covariates, etc. Some studies used different units of measurement for IL-6, but we transformed these units into pg/mL for clarity. Studies in which IL-6 was transformed into a logarithmic scale were analyzed separately, to appropriately account for methodological differences. A transformation back to a common unit would complicate direct comparisons and pooling of results.

Data on plasma IL-6 values at hospital admission and subsequent measurements were carefully noted and analyzed, along with their associated timings (e.g., 24, 48 h). We prioritized corresponding effect measures from multivariable models, focusing on adjusted odds ratios (ORs) and hazard ratios (HRs). These OR and HR values were often reported at multiple timepoints throughout the studies, and any provided area under the receiver operating characteristics (AUROC) curves to determine outcomes related to baseline and follow-up intervals were captured.

The primary outcome of this meta-analysis was mortality, specifically, all-cause mortality, sepsis-related mortality, and in-hospital mortality, with a particular focus on the 28–30-day timeframe. This widely accepted clinical endpoint captures both early deaths associated with the acute phase of sepsis, and those occurring due to later complications or persistent organ dysfunction. All included studies reported mortality data either as in-hospital mortality or within the specified 28–30-day follow-up period, allowing for standardized assessment of short-term outcomes and the prognostic value of interleukin-6 (IL-6). Based on this outcome, patients were classified into two groups: survivors (S) and non-survivors (NS), facilitating analysis of IL-6’s potential impact on survival.

### 2.4. Risk of Bias Assessment

We assessed the risk of bias within our included studies using the Quality In Prognosis Studies (QUIPS) tool [17], tailored specifically for our review (Supplemental Materials, Tables S2 and S3). The QUIPS tool evaluates six key domains: study participation (D1), study attrition (D2), prognostic factor measurement (D3), outcome measurement (D4), confounding measurement (D5), and statistical analysis and reporting (D6). Two reviewers independently scored each study for risk of bias across all domains, resolving discrepancies through discussion with a third expert. As suggested by the tool guidelines, each study was then assigned a “low”, “moderate”, or “high” risk of bias rating for each domain. In the confounding domain (D5), while acknowledging that different studies adjusted for diverse covariates (most often other biomarkers like procalcitonin, lactate, WBC, severity scores, body mass index, alcohol dependence, cancer, etc.), our expert group focused on age and at least one severity score (SOFA or APACHE-II) as critical and consistent adjustment factors for this review.

### 2.5. Statistical Analysis

To evaluate the association between baseline IL-6 levels and 28-day mortality, we performed two types of meta-analyses. The first type was a meta-analysis of effect measures: we pooled the odds ratios (ORs), hazard ratios (HRs), and risk ratios (RRs) reported in the included studies. Where available, we prioritized effect measures derived from multivariable models that adjusted for potential confounders. The second type was a meta-analysis of the area under the receiver operating characteristics (AUROC) curve: we pooled the AUROC values to assess the discriminatory ability of baseline IL-6 levels for predicting 28-day mortality.

For IL-6 kinetics within the first 96 h after admission, we also conducted meta-analyses of both effect measures and AUROC values, using the same approach as described above. In all meta-analyses, we employed a random effects restricted maximum likelihood (REML) model to account for heterogeneity between studies. Pooled estimates were calculated using the Sidik–Jonkman method with Hartung–Knapp 95% confidence intervals. Heterogeneity was assessed using the I2 statistic and Cochran’s Q test. Publication bias was evaluated using Egger’s and Begg’s tests.

All meta-analyses were performed using MedCalc software for Windows, version 23.1.7 (MedCalc Software, Ostend, Belgium).

## 3. Results

### 3.1. Overview of Selected Studies

The initial search of the electronic databases, using the aforementioned keywords, resulted in 22,337 studies in total. An initial examination of titles led to the exclusion of 8149 duplicates and 12,931 studies with other research aims (not evaluating the prognostic value of IL-6 in sepsis). After a thorough examination of the abstracts of the remaining studies, a total of 1208 studies were excluded because they did not serve our study objectives (*n* = 743), used unclear definitions for sepsis (*n* = 48), or employed other study designs (*n* = 407), such as reviews, case reports, etc. The remaining articles were assessed for eligibility through full-text review. The studies that were excluded [17,18,19,20,21,22,23,24,25,26,27,28,29,30,31,32,33] from the meta-analysis after the final screening step, and the reasons for exclusion, are described in the Supplemental Materials, Table S1.

Our selection process resulted in a final set of 31 studies [34,35,36,37,38,39,40,41,42,43,44,45,46,47,48,49,50,51,52,53,54,55,56,57,58,59,60,61,62,63,64], including a total of 4566 septic patients. Out of these 31 studies, 16 investigated the fluctuations in subsequent (i.e., post-baseline) IL-6 measurements, and the corresponding measure of effect on the outcome. All 31 studies used mortality as the primary outcome, with slight variations: 25 studies examined 28–30-day mortality periods, while 4 focused on in-hospital mortality without specific timelines. One study reported outcomes at 4-day, 28-day, and 1-year timepoints, but only the first 28 days were accounted for in the meta-analysis [30]. Publication trends revealed that 3 articles were published between 2000 and 2010, 17 between 2010 and 2020, and 11 between 2020 and 2023. Most studies were performed in China, encompassing 10 of the included studies, followed by Spain, with 4, and South Korea, Greece, and Japan, with 2. Of the included studies, 24 were prospective, with 7 utilizing a retrospective design. The clinical settings in which the studies were performed were mostly ICU wards (22 studies), while 5 studies were performed in the ER, 3 studies were performed in mixed clinical settings (ICU + a medical ward), and 1 study was performed in the Department of Traumatology and Acute Critical Medicine. The definitions of sepsis differed greatly across studies, reflecting their publication dates: 18 used the Singer 2015 criteria, 10 employed the Levy 2001 criteria, and 3 used other well-defined criteria. A general overview of the selected studies is provided in Table 1.

The number of patients included in the studies ranged from 29 to 708, with a median of 93 (25th–75th percentiles 62.5–174). From a total of 4566 patients, the percentage of men was 62.58% on average, ranging across studies from 43.8% to 81.8%. Age reporting varied across studies, with all providing either mean or median values. In 12 studies, age was reported separately for the two groups of patients, survivors (S) vs. non-survivors (NS). Twenty-six studies reported a mixed origin of sepsis in their patients, one article focused exclusively on patients with sepsis of respiratory origin, and four studies did not report the origin of sepsis.

### 3.2. Risk of Bias

Across all studies, the QUIPS domain with the highest risk of bias was statistical analysis and reporting (domain 6), for which 15/31 studies received a “high” rating. The reasons for this were as follows: reporting exclusively on statistically significant values, limitations in describing statistical methodologies, or underutilizing available data (for instance, some articles failed to report information such as the area under the receiver operating characteristics curve to determine outcomes, IL-6 cut-off values, sensibility, specificity, positive and negative predictive values, etc.). If two or more items were considered problematic, a “high” rating was given. Seven articles received a “moderate” rating, reflecting only one of the aforementioned concerns, and nine studies received a “low” rating. Confounding measurements (Domain 5) presented challenges in assessment because of the wide variation in prognostic confounders across studies. The two most frequent covariates were age and at least one severity score (APACHE-II or SOFA), justifying our decision to use these as the main covariates. Studies failing to adjust for both at a minimum received a “high” risk of bias rating in Domain 5. Overall, the remaining domains of the QUIPS tool posed a low-to-moderate risk of bias across the majority of the studies. A complete breakdown of our tailored application of the QUIPS domains, along with the rationale used for every assessment, is provided in the Supplemental Materials, Tables S2 and S3.

### 3.3. Meta-Analysis

A comprehensive data extraction table was constructed for this study, documenting study ID, SOFA and APACHE-II scores, baseline IL-6 levels, IL-6 clearance at various timepoints, and effect estimates from IL-6 logistic regression and AUROC analyses (with 95% confidence intervals). All the data that were gathered from each individual article and used for the meta-analysis can be found in Table S4.

As illustrated in Table 2, among the 31 studies, 12 reported the association between IL-6 levels and mortality using odds ratios (ORs), with a total of 2004 patients. Five studies reported hazard ratios (HRs), encompassing 461 patients. One study with 708 patients reported risk ratios (RRs). Twelve studies did not report suitable effect estimates for inclusion in the meta-analysis. Two studies employed log-transformed ORs, including data from 284 patients.

Regarding model adjustments, 13 studies accounted for both age and disease severity, with 7 utilizing the SOFA score, 7 using APACHE-II, and 5 incorporating both. Age was the most frequently adjusted-for confounder (15 studies), followed by SOFA (12 studies) and APACHE-II (11 studies). Nineteen studies included adjustments for various biomarkers. Notably, studies adjusting for both age and severity tended to report higher odds ratios (median OR 1.13) compared to those adjusting for age alone (median OR 1.033) or not adjusting for age (median OR 1.002), suggesting that these factors may confound the relationship between IL-6 and mortality.

#### 3.3.1. Baseline IL-6 Values and IL-6 Clearance: Effect Measures

**Figure 2:**
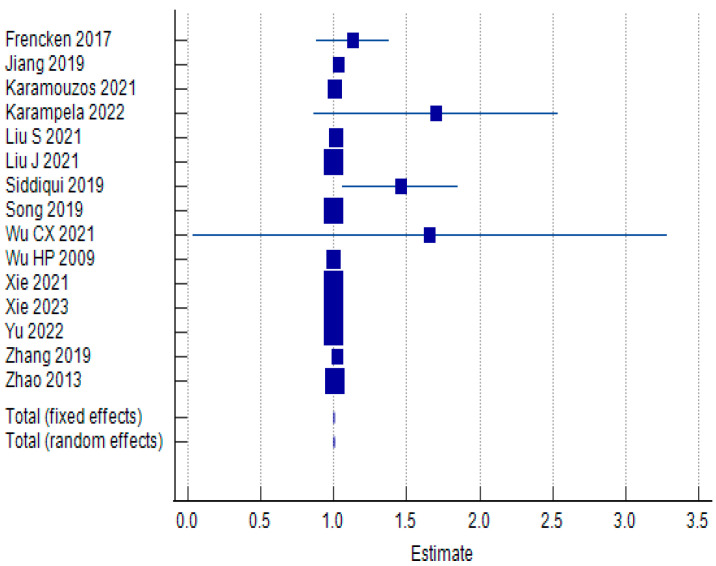
Forest plot examining association between baseline IL-6 values and 28–30-day mortality in sepsis patients using random effects model [38,40,41,42,43,44,51,52,58,59,60,61,62,63,64].

The overall pooled odds ratio at baseline (OR) is 1.001, suggesting that higher baseline IL-6 levels are not associated with a significant increase in mortality risk. The 95% confidence interval (CI) for the pooled OR is very narrow, ranging from 0.999 to 1.003. This further supports the conclusion of no statistically significant association between baseline IL-6 levels and mortality. The forest plot can be observed in Figure 2, while the results of the meta-analysis can be seen in Table 3.

The test for heterogeneity (Q = 31.8881, *p* = 0.0042) reveals significant heterogeneity between the studies included in the meta-analysis. This suggests that factors other than chance contribute to the differences in results across studies, such as variations in study design, patient populations, or IL-6 measurement methods. The I^2^ statistic (56.10%) confirms moderate heterogeneity. Egger’s test shows a significant *p*-value (*p* = 0.0005), suggesting potential publication bias. This means that studies with positive or significant results might be more likely to be published, leading to an overestimation of the true effect in the meta-analysis. Begg’s test is also significant (*p* = 0.0234), further supporting the presence of publication bias.

**Figure 3:**
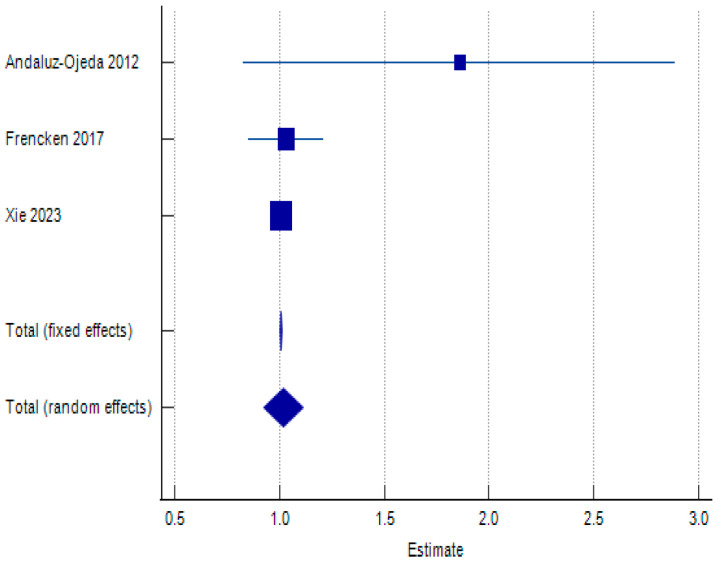
Forest plot examining association between IL-6 levels within first 96 h after admission (IL-6 0–96 h) and 28–30-day mortality in sepsis patients using random effects model [34,38,61].

Regarding IL-6 clearance in the 96 h timeframe, the overall pooled OR is 1.019, indicating that elevated IL-6 levels in the first 96 h after admission are not associated with a statistically significant increase in mortality risk. The 95% confidence interval (CI) for the pooled OR ranges from 0.925 to 1.112. Since this interval includes 1, we cannot be confident that the true effect is different from no effect. The results of the meta-analysis can be found in Table 4, with the respective forest plot in Figure 3.

The test for heterogeneity (Q = 2.7022, *p* = 0.2590) reveals no significant heterogeneity between the three studies included in the meta-analysis. This implies that the observed differences in results across studies are likely due to chance, rather than systematic differences in study design or population. The I^2^ statistic (25.99%) indicates low heterogeneity. Egger’s test shows a non-significant *p*-value (*p* = 0.3970), suggesting no significant evidence of publication bias based on this test. Begg’s test is also not significant (*p* = 0.1172), further supporting the absence of publication bias. Based on these results, we cannot conclude that IL-6 levels within the first 96 h after admission have significant prognostic value for predicting mortality in sepsis patients using logistic regression. Only three studies could be used to perform this test, which limits the power to detect a small but clinically significant effect.

In a separate analysis of two studies reporting log-transformed odds ratios (284 patients), a significant association was found between elevated IL-6 levels and mortality in sepsis patients. At admission, a one-unit increase in log IL-6 was associated with an 81% increase in the odds of mortality (pooled log OR 1.81, 95% CI 1.44–2.27). This association persisted on day 3, with a one-unit increase in log IL-6 corresponding to a 165% increase in the odds of mortality (pooled log OR 2.65, 95% CI 1.99–3.53). These findings suggest that IL-6 may be a valuable prognostic marker in this subset of studies, although the generalizability of these results to the broader population of sepsis patients is limited by the small number of studies included in this analysis and the methodological differences in reporting effect estimates. Due to the inclusion of only two studies, formal assessment of heterogeneity and publication bias was not conducted.

#### 3.3.2. Area Under the Receiver Operating Characteristic Curve

**Figure 4:**
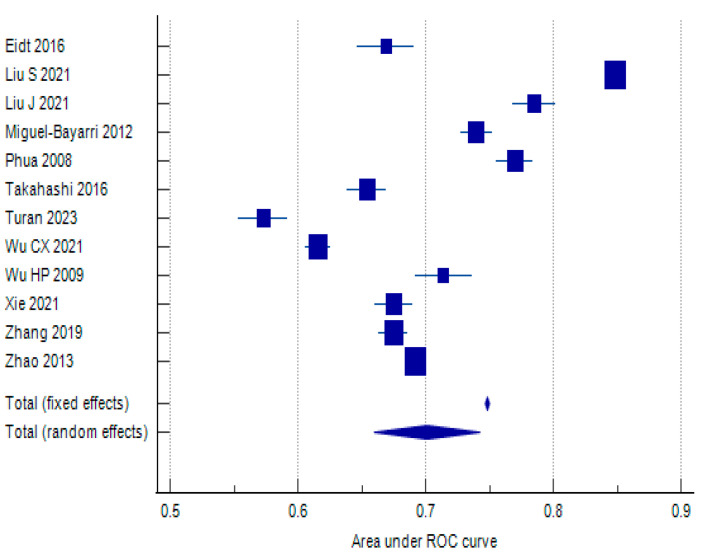
Forest plot assessing discriminatory ability of baseline IL-6 plasma values for predicting 28–30-day mortality [37,43,44,46,48,53,55,58,59,60,63,64].

The overall pooled AUROC is 0.701 (95% CI: 0.660–0.742), indicating that the ability of baseline IL-6 levels to predict 28-day mortality in sepsis patients is moderate. The test for heterogeneity (Q = 8874.2114, *p* < 0.0001) reveals significant heterogeneity between the studies. This means that the differences in AUROC values between studies are likely due to factors other than chance, such as differences in study design, patient populations, or IL-6 measurement methods. The I^2^ statistic (99.88%) further confirms the substantial heterogeneity. The results of the statistical testing can be found in Table 5, with the respective forest plot in Figure 4.

Egger’s test shows a non-significant *p*-value (*p* = 0.4378), indicating no significant evidence of publication bias based on this test. Begg’s test is also not significant (*p* = 0.4929), further supporting the absence of publication bias. Based on these results, baseline IL-6 appears to have a moderate ability to predict mortality in sepsis patients. However, the significant heterogeneity between studies suggests that the predictive performance of IL-6 may vary depending on various factors.

**Figure 5:**
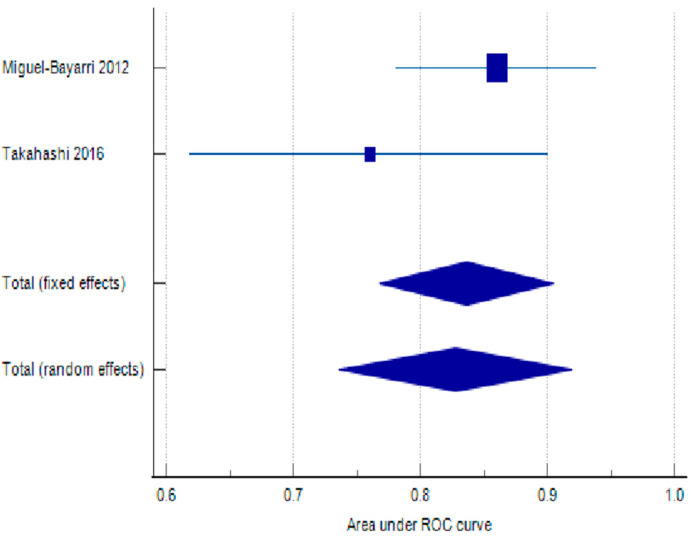
Forest plot assessing discriminatory ability of IL-6 clearance at 96 h post admission [46,53].

Regarding IL-6 clearance, the overall pooled AUROC is 0.828 (95% CI: 0.736–0.919), indicating that IL-6 levels within the first 96 h have good discriminatory power for predicting mortality in sepsis patients. The test for heterogeneity (Q = 1.4741, *p* = 0.2247) shows no significant heterogeneity between the two studies included in the meta-analysis. This implies that the observed differences in results across studies are likely due to chance, rather than systematic differences in study design or population. The I^2^ statistic (32.16%) with a confidence interval spanning from 0.00 to 100.00 also reflects the uncertainty due to the small number of studies. The results of the statistical testing can be found in Table 6, with the respective forest plot in Figure 5.

Egger’s test reveals a significant *p*-value (<0.0001), suggesting potential publication bias. This means that studies with positive or significant results might be more likely to be published, leading to an overestimation of the true effect in the meta-analysis. Begg’s test, however, is not significant (*p* = 0.3173), indicating no significant evidence of publication bias based on this test alone. These results imply that IL-6 levels within the first 96 h after admission appear to have good potential as a predictor of mortality in sepsis patients. However, the confidence interval for the pooled AUROC is relatively wide (0.736 to 0.919), which suggests that there is still considerable uncertainty regarding the true discriminatory power of IL-6, due to the limited number of studies included.

## 4. Discussion

This systematic review and meta-analysis sought to clarify the prognostic value of interleukin-6 (IL6) in predicting mortality among critically ill patients with sepsis. Our investigation, encompassing both baseline IL-6 levels and their dynamic changes over time, revealed a nuanced picture. While traditional effect measures like odds ratios (ORs) and hazard ratios (HRs) did not demonstrate a statistically significant association between IL-6 and mortality, analyses of the area under the receiver operating characteristic curve (AUROC) unveiled the cytokine’s potential as a discriminatory tool. This suggests that IL-6, though perhaps not a potent independent predictor of mortality, may, nonetheless, possess value in differentiating between patients at higher and lower risk of death, a finding with potential implications for risk stratification and personalized therapeutic strategies.

Our observation that baseline IL-6 levels are not significantly associated with increased mortality risk aligns with the findings of Molano-Franco et al. [65], who similarly reported no significant association between the baseline values of several biomarkers, including IL-6, and mortality in septic patients. However, our study differs in that it focused exclusively on IL-6, and incorporated a larger number of studies (*n* = 31) and patients (*n* = 4566) than the Molano-Franco et al. analysis. Additionally, our work uniquely examined the prognostic value of IL-6 kinetics, analyzing changes in IL-6 levels over the first 96 h, whereas the Molano-Franco et al. study primarily evaluated baseline values. This expanded scope offers a more comprehensive assessment of IL-6’s potential role as a prognostic biomarker in sepsis. In contrast to the Li et al. [66] meta-analysis, which examined cytokine changes after treatment initiation, our study specifically examined the prognostic potential of IL-6 dynamics within the first 96 h, independently of therapeutic interventions. These findings, along with our own, highlight the complexities of using IL-6 as a single prognostic indicator in sepsis, and suggest the need for a more nuanced approach.

The concept of biomarker clearance as a predictor of outcomes in sepsis patients has been explored with other inflammatory markers as well, particularly procalcitonin (PCT), C-Reactive Protein (CRP), lactate (LAC), etc. Researchers have sought to determine whether a significant drop in the plasma levels of these markers, within a certain timeframe, is associated with better outcomes. For instance, Charles et al. (2009) found that a PCT decrease of >30% before the third day of hospitalization was an independent predictor of survival in ICU patients with sepsis [67]. Similarly, Karlsson et al. (2010) reported lower mortality in patients with a >50% decrease in PCT by 72 h [68]. Other studies have also demonstrated the prognostic value of PCT and CRP clearance, with varying definitions and timeframes [69,70,71,72,73].

The divergence between our findings based on pooled effect measures, such as odds ratios (ORs) and hazard ratios (HRs), and those derived from the area under the receiver operating characteristic curve (AUROC) merits further exploration. Odds ratios and hazard ratios estimate how strongly IL-6 levels predict mortality risk—OR reflects the odds of death with higher IL-6, while HR tracks risk over time—yet our results showed no significant link, suggesting that any risk increase is minor or negligible across a broad patient group. A key complicating factor is the wide variability in IL-6 levels observed in sepsis. In our clinical studies, IL-6 concentrations spanned a broad range, which could have reduced the reliability of statistical tests like OR and HR by introducing inconsistency that diluted their ability to detect a clear pattern. Even in preclinical research with tightly controlled conditions, such as the mouse model study by Lee et al. (2022), IL-6 levels fluctuated significantly following an inflammatory trigger, indicating that this variability is an inherent biological trait rather than a measurement artifact [74]. This natural fluctuation likely weakened our pooled OR and HR estimates, explaining their lack of statistical significance despite IL-6’s moderate predictive strength in AUROC analyses. In contrast, the AUROC assesses how well IL-6 distinguishes between patients who survive and those who do not, regardless of the exact risk magnitude. Our moderate-to-good AUROC values for baseline and early IL-6 measurements suggest that it can still flag higher-risk patients effectively. This contrast highlights a critical point: a lack of statistical significance in risk estimates does not necessarily diminish clinical utility. Though IL-6 may not sharply elevate mortality odds, its capacity to stratify risk could still inform patient management decisions.

Our findings are complicated by the considerable heterogeneity we observed across the studies we analyzed. This variation stems from a multitude of factors related to the patient populations of our included studies: the severity of sepsis, IL-6 measurement techniques, and statistical methodologies. Notably, the variability in sepsis definitions across the included studies, ranging from older criteria (Levy 2001) [75] to more recent ones (Singer 2015) [1], could introduce heterogeneity in the assessment and prognosis of sepsis. Our findings must also be considered in the context of aging, which significantly shapes IL-6 dynamics. According to Ershler and Keller [76], aging drives elevated baseline IL-6 levels through chronic low-grade inflammation—termed “inflammaging”—potentially reducing its specificity as a prognostic marker in older septic patients. Similarly, Ferrucci et al. [77] highlighted that this persistent IL-6 elevation in the elderly correlates with frailty and immune dysregulation, which could mask acute sepsis-related spikes. A study by Puzianowska-Kuźnicka et al. [78] further demonstrated that age-related shifts in IL-6 receptor signaling, especially in T cells, impair immune responses, possibly contributing to the heterogeneity we observed across studies with diverse median ages. These insights emphasize the need for age-stratified analyses in future research to unravel IL-6’s prognostic role across the lifespan.

Furthermore, the potential for publication bias, as indicated by some of our analyses, warrants consideration. While we have strived to include a comprehensive range of studies, it is possible that studies with positive or significant findings were more likely to be published, potentially leading to an overestimation of the true effect. This observation emphasizes the necessity for careful interpretation of data, and highlights the importance of future research that addresses these limitations.

Beyond age, genetic variability adds another layer to IL-6’s prognostic complexity. Single-nucleotide polymorphisms (SNPs) in genes like IL-10, an anti-inflammatory cytokine, can shift its activity [79,80]. Overactive variants might dampen IL-6’s inflammatory drive, while underactive ones could let it run unchecked, altering plasma levels and their link to mortality. Our meta-analysis, built on summary data, could not tease out these effects, but they likely fueled some of the heterogeneity we saw across studies. This suggests that genetic profiles could muddy IL-6’s signal, a wrinkle worth exploring in future work. IL-6 itself carries genetic variability. As a member of a cytokine family steering acute-phase responses, IL-6’s gene on chromosome 7p21 harbors variants like the −174 C/G promoter polymorphism [28]. The GG genotype boosts IL-6 production over the CC, potentially intensifying inflammation and mortality risk in sepsis [28,81]. Without genotype-specific data, our analysis could not probe this, but it is a plausible driver of IL-6’s uneven prognostic strength, highlighting the urgency of future genotype-focused studies.

Our study has several noteworthy limitations. Firstly, despite including a substantial number of studies that investigated the role of IL-6 in sepsis prognosis, there was a limited number of studies that reported the dynamic changes in plasma IL-6 over time. The significant heterogeneity observed across the included studies also introduces a degree of uncertainty into our conclusions. These factors limit the generalizability of our findings. Secondly, our focus on 28–30-day mortality, while reflecting the predominant trend in sepsis research, may have inadvertently excluded valuable insights from studies examining longer-term outcomes, such as 90-day mortality. These longer-term outcomes, however, might be significantly influenced by factors that are not related to sepsis (e.g., old age, comorbidities, complications, etc.). These confounding factors can make it challenging to isolate the specific contribution of any specific biomarker to mortality risk over extended periods. Thirdly, the generalizability of our findings might also be affected by the potential for bias in the primary studies, variability in patient characteristics, and the exclusion of certain populations (patients under the age of 18, or immunocompromised individuals). Lastly, relying on a single biomarker like IL-6 to predict mortality in a complex syndrome like sepsis has inherent limitations. Sepsis is a multifaceted phenomenon, driven by a complex interplay of pathophysiological processes, and a single molecule does not adequately capture this complexity. Even if our findings suggest a limited independent prognostic value for IL-6, our systematic review and meta-analysis provides future research suggestions and prevents redundant efforts in the future.

Moving forward, research should prioritize addressing the limitations identified in this review and exploring new avenues for understanding the role of IL-6 in sepsis prognosis. Future studies should delve deeper into the sources of heterogeneity observed in IL-6 studies, potentially employing techniques like meta-regression to analyze the relationship between study characteristics and effect sizes. Given the limitations of single biomarkers, the development and validation of multi-marker panels that incorporate IL-6 alongside other promising candidates, such as procalcitonin, CRP, and lactate, hold immense potential for improving prognostic accuracy. This could be further enhanced by leveraging machine learning techniques to identify optimal biomarker combinations and generate more precise risk stratification models. To address the limitations of relying on summary data, future research should prioritize collecting and analyzing individual patient data. This would allow for a more granular investigation of IL-6 kinetics, including the determination of optimal IL-6 thresholds for predicting mortality and the exploration of potential interactions with other biomarkers. Furthermore, dedicated studies are needed to investigate the prognostic value of IL-6 in specific sepsis subpopulations, such as immunosuppressed patients, pediatric patients, and those with different infection sources or pathogens.

## 5. Conclusions

In conclusion, while our systematic review and meta-analysis suggest that IL-6, whether measured at baseline or within the first 96 h after admission, may not be a reliable independent predictor of mortality in critically ill patients with sepsis, the moderate-to-good discriminatory ability of IL-6 observed in the AUROC analyses warrants further investigation. Future research should focus on identifying sources of heterogeneity, exploring the impact of different IL-6 thresholds, and considering the use of IL-6 as part of a multi-marker panel, or in specific patient subgroups, to unlock its potential in refining sepsis prognosis and guiding personalized treatment decisions.

## Figures and Tables

**Table 1:** Overview of included studies; ICU = intensive care unit; S = survivors; NS = non-survivors; SD = standard deviation; NR = not reported; DTACM = Department of Traumatology and Acute Critical Medicine.

Study ID	Country	Clinical Setting	Patient Recruitment	N	Events/N	Age (years)	Sex (% of Males)	Outcome	Sepsis Criteria	Sepsis Origin
Andaluz-Ojeda 2012 [34]	Spain	ICU	Prospective	29	12/29	66.1; mean	58.70%	28–30-day mortality	Levy 2001	Mixed
Belli 2022 [35]	Italy	ICU	Prospective	35	15/35	59 (48–60); median (25th–75th)	60%	28–30-day mortality	Singer 2015	Mixed
Beneyto 2016 [36]	Spain	ICU	Prospective	203	52/203	65; mean	64%	In-hospital mortality	Levy 2001	Mixed
Eidt 2016 [37]	Brazil	ICU	Prospective	48	21/48	61.4 (18.7) in severe sepsis; 65.9 (17.8) in septic shock; mean (SD)	50%	ICU mortality	Levy 2001	NR
Frencken 2017 [38]	Netherlands	ICU	Prospective	708	226/708	63 (53–72); median (25th–75th)	60%	4-day, 28-day, and 1-year mortality	Singer 2015	Mixed
Jekarl 2013 [39]	South Korea	Mixed	Prospective	78	15/78	62.1 (19.9); mean (SD)	45%	28–30-day mortality	Levy 2001	Mixed
Jiang 2019 [40]	China	ICU	Prospective	198	88/198	69.9 (28–91) in S vs. 68.7 (18;91) in NS; median (25th–75th)	81.80%	28–30-day mortality	Singer 2015	Mixed
Karamouzos 2021 [41]	Greece	ICU	Retrospective	128	46/128	72.4 (15) in S vs. 77.3 (10) in NS; mean (SD)	43.80%	28–30-day mortality	Singer 2015	Mixed
Karampela 2022 [42]	Greece	ICU	Prospective	102	30/102	64.7 (15.6); mean (SD)	55.90%	28–30-day mortality	Singer 2015	Mixed
Liu S. 2021 [43]	China	ICU	Retrospective	264	78/264	52.9 (12.6); mean (SD)	64%	28–30-day mortality	Singer 2015	Mixed
Liu J. 2021 [44]	China	ICU	Prospective	66	17/66	69 (54–82) in S vs. 77 (63–84) in NS; median (IQR)	59%	28–30-day mortality	Singer 2015	Mixed
Matsumoto 2018 [45]	Japan	DTACM	Retrospective	31	7/31	73.0 (65.0–81.0); median (IQR)	74%	28–30-day mortality	Singer 2015	Mixed
Miguel-Bayarri 2012 [46]	Spain	ICU	Prospective	81	27/81	62, median; no other details	54%	28–30-day mortality	Levy 2001	Mixed
Oberholzer 2005 [47]	USA	Mixed	Prospective	124	39/124	58.3 (17.5); mean (SD)	55%	28–30-day mortality	Other	NR
Phua 2008 [48]	Singapore	ICU	Prospective	72	30/72	55 (16) in S vs. 54 (17) in NS; mean (SD)	64%	28–30-day mortality	Levy 2001	Mixed
Ricarte-Bratti 2017 [49]	Argentina	ICU	Prospective	48	21/48	53.8 (17.1) in S vs. 73.2 (8.9) in NS; mean (SD)	46%	28–30-day mortality	Singer 2015	Mixed
Rios-Toro 2017 [50]	Spain	ICU	Prospective	50	21/50	68 (53–75); median (25th–75th)	72%	28–30-day mortality	Levy 2001	Mixed
Siddiqui 2019 [51]	Singapore	ICU	Prospective	198	NR	63.0 (49.5;73.5); 54.0 (45.0;64.0); 61.0 (40.0;68.8); mean (SD)	60.10%	28–30-day mortality	Other	NR
Song 2019 [52]	South Korea	ER	Prospective	97	NR	75 (42;98); median (25th–75th)	56.00%	28–30-day mortality	Singer 2015	Mixed
Takahashi 2016 [53]	Japan	ICU	Prospective	85	15/85	68 (59–77); median (25th–75th)	62.40%	28–30-day mortality	Singer 2015	Mixed
Thao 2018 [54]	Vietnam	ICU	Prospective	123	75/123	62 (46–75) in S; 54 (43–73) in NS; median (25th–75th)	NR	In-hospital mortality	Levy 2001	Mixed
Turan 2023 [55]	Turkey	ICU	Prospective	60	39/60	60 (56–68) in S vs. 74 (62–80) in NS; median (IQR)	60.00%	28–30-day mortality	Singer 2015	Mixed
Vivas 2021 [56]	Colombia	ICU	Prospective	62	10/62	53 (19.47); mean (SD)	59.67%	In-hospital mortality	Singer 2015	Mixed
Weidhase 2019 [57]	Germany	ICU	Retrospective	328	118/328	64 [54;73] in S vs. 62 [55;69] in NS; median (IQR)	63.10%	In-hospital mortality	Levy 2001	Mixed
Wu C.X. 2021 [58]	China	ICU	Prospective	114	51/114	71 (60;81); median (25th–75th)	72.90%	28–30-day mortality	Singer 2015	Mixed
Wu H.P. 2009 [59]	China	Mixed	Prospective	63	14/63 (22.2%)	70.0 (2.0) in S vs. 69.1 (4.1) in NS; mean (SD)	63.50%	28–30-day mortality	Other	Respiratory
Xie 2021 [60]	China	ER	Retrospective	90	23/90	72 (26–97) in S vs. 77 (50–97) in NS; median (25th–75th)	64%	28–30-day mortality	Singer 2015	Mixed
Xie 2023 [61]	China	ER	Retrospective	367	53/367	71 (19–98) in S vs. 80 (46–97) in NS; median (25th–75th)	65.90%	28–30-day mortality	Singer 2015	Mixed
Yu 2022 [62]	China	ER	Prospective	63	NR	79 (34–95); median (25th–75th)	63.50%	28–30-day mortality	Singer 2015	NR
Zhang 2019 [63]	China	ICU	Retrospective	150	16/66 in sepsis group and 48/84 in septic shock group	70 (24–91) in sepsis group vs. 74.5 (24–89) in septic shock group; median (25th–75th)	63.40%	28–30-day mortality	Singer 2015	Mixed
Zhao 2013 [64]	China	ER	Prospective	501	134/504	73 (58–79) in S vs. 77 (65–83) in NS; median (IQR)	55.70%	28–30-day mortality	Levy 2001	Mixed

**Table 2:** IL-6 values and mortality: effect measures. HR = hazard ratio; OR = odds ratio; D = day; NR = not reported.

Study ID	N	Events/N	IL-6 Values and Mortality: Effect Measures (95% CI)	Model Adjusted by
Andaluz-Ojeda 2012 [34]	29	12	Adjusted HR at D3: 1.86 (1.08–3.20); D28: 2.00 (1.22−3.27)	APACHE II, other biomarkers
Belli 2022 [35]	35	15	HR = 1.000 (1.000–1.000)	Univariable
Beneyto 2016 [36]	203	52	Admission: log IL-6 OR = 1.62 (1.24–2.13); D3: log IL-6 OR = 2.69 (1.64–4.40)	Age, sex, APACHE II, SOFA, other biomarkers
Eidt 2016 [37]	48	21	NR	Age, sex, lactate, other biomarkers
Frencken 2017 [38]	708	226	Admission: RR = 1.13 (0.91–1.41); D4: RR = 1.03 (0.86–1.23)	Age, Charlson comorbidity index, immunodeficiency, site of infection
Jekarl 2013 [39]	78	15	NR	Other biomarkers
Jiang 2019 [40]	198	88	OR = 1.033 (1.007–1.061)	SOFA, other biomarkers such as EPO, hepcidin, ferritin, sTfR/log ferritin
Karamouzos 2021 [41]	128	46	OR = 1.002 (0.996–1.008)	Microorganism, MDR status, type of infection, cytokines
Karampela 2022 [42]	102	30	HR = 1.70 (1.05–2.74)	Age, Sex, BMI, APACHE-II, presence of septic shock
Liu S. 2021 [43]	264	78	OR = 1.017 (1.005–1.028)	Age, sex, BMI, SBP, APACHE-II, SOFA
Liu J. 2021 [44]	66	17	OR = 1.001 (1.000–1.001)	Age, SOFA, other biomarkers
Matsumoto 2018 [45]	31	7	Maximum values from three days (D1, D2, D4): 19.62 (3.47–110.80)	SOFA
Miguel-Bayarri 2012 [46]	81	27	Admission: log IL-6 OR = 1.98 (1.27–3.09); D3: log IL-6 OR = 2.6 (1.43–4.71)	Age, sex, development of MOF, other biomarkers, SOFA, APACHE-II
Oberholzer 2005 [47]	124	39	Not significant (*p*-value)	APACHE-II, age, treatments, baseline MOD
Phua 2008 [48]	72	30	NR	APACHE-II
Ricarte-Bratti 2017 [49]	48	21	NR	Univariable
Rios-Toro 2017 [50]	50	21	NR	Age, SOFA, APACHE II, other biomarkers
Siddiqui 2019 [51]	198	NR	HR = 1.46 (1.11−1.92)	Age, sex, surgical methods, qSofa
Song 2019 [52]	97	NR	HR = 1.001 (1.000–1.002)	APACHE-II, SOFA, pentraxin, lactate
Takahashi 2016 [53]	85	15	NR	Age, sex, SOFA
Thao 2018 [54]	123	75	IL-6 clearance in 24 h: ≥86%, OR = 5.67 (1.27–25.3); Il-6 clearance between 85% and 50%, OR = 1.86 (0.44–7.94)	Age, sex, BUN, Creatinine, aPTT, pH
Turan 2023 [55]	60	39	NR	Age, sex, diagnosis, SOFA, APACHE II, other biomarkers
Vivas 2021 [56]	62	10	NR	Age, site of infection
Weidhase 2019 [57]	328	118	NR	NR
Wu C.X. 2021 [58]	114	51	OR = 1.66 (0.67−4.10)	SOFA and IL-37
Wu H.P. 2009 [59]	63	14	OR = 1.00 (0.99−1.01)	APACHE-II, septic shock, gastrointestinal bleeding, IL-10 and TGF-β1
Xie 2021 [60]	90	23	OR = 1.000 (1.000–1.001)	Lactate, neutrophil-to-WBC ratio

**Table 3:** Study-specific and pooled effect estimates of association between baseline IL-6 levels and mortality; CI = confidence interval.

Study	Estimate	Standard Error	Lower Limit of 95% CI	Upper Limit of 95% CI	z	*p*-Value	Weight (%)—Fixed	Weight (%)—Random
Frencken 2017 [38]	1.13	0.127	0.881	1.379			0.001	0.004
Jiang 2019 [40]	1.033	0.014	1.006	1.060			0.082	0.33
Karamouzos 2021 [41]	1.002	0.003	0.996	1.008			1.79	5.32
Karampela 2022 [42]	1.7	0.427	0.863	2.537			0.009	0.004
Liu S. 2021 [43]	1.017	0.006	1.005	1.029			0.45	1.68
Liu J. 2021 [44]	1.001	0.001	0.999	1.003			16.14	14.95
Siddiqui 2019 [51]	1.46	0.202	1.064	1.856			0.001	0.001
Song 2019 [52]	1.001	0.001	0.999	1.003			16.14	14.95
Wu C.X. 2021 [58]	1.66	0.828	0.0371	3.283			0.001	0.001
Wu H.P. 2009 [59]	1	0.005	0.99	1.01			0.65	2.33
Xie 2021 [60]	1	0.001	0.998	1.002			16.14	14.95
Xie 2023 [61]	1	0.001	0.998	1.002			16.14	14.95
Yu 2022 [62]	0.999	0.001	0.997	1.001			16.14	14.95
Zhang 2019 [63]	1.02	0.01	1	1.04			0.16	0.64
Zhao 2013 [64]	1.002	0.001	1	1.004			16.14	14.95
Total (fixed effects)	1.001	0	1	1.001	2490.434	<0.001	100	100
Total (random effects)	1.001	0.001	0.999	1.003	1230.89	<0.001	100	100

**Table 4:** Study-specific and pooled effect estimates of association between IL-6 clearance and mortality; CI = confidence interval.

Study	Estimate	Standard Error	Lower Limit of 95% CI	Upper Limit of 95% CI	z	*p*-Value	Weight (%)—Fixed	Weight (%)—Random
Andaluz-Ojeda 2012 [34]	1.86	0.525	0.831	2.889			0.001	0.82
Frencken 2017 [38]	1.03	0.092	0.85	1.21			0.047	20.12
Xie 2023 [61]	1.007	0.002	1.003	1.011			99.95	79.06
Total (fixed effects)	1.007	0.002	1.003	1.011	503.634	<0.001	100	100
Total (random effects)	1.019	0.048	0.925	1.112	21.316	<0.001	100	100

**Table 5:** Discriminatory ability of baseline IL-6 values, according to area under receiver operating characteristics.

Study	ROC Area	Standard Error	Lower Limit of 95% CI	Upper Limit of 95% CI	Z	*p*-Value	Weight (%)—Fixed	Weight (%)—Random
Eidt 2016 [37]	0.669	0.0115	0.646	0.692			0.54	8.22
Liu S. 2021 [43]	0.849	0.0014	0.846	0.852			36.72	8.42
Liu J. 2021 [44]	0.785	0.0086	0.768	0.802			0.97	8.31
Miguel-Bayarri 2012 [46]	0.74	0.0065	0.727	0.753			1.70	8.36
Phua 2008 [48]	0.77	0.0072	0.756	0.784			1.39	8.34
Takahashi 2016 [53]	0.654	0.0078	0.639	0.669			1.18	8.33
Turan 2023 [55]	0.573	0.0098	0.554	0.592			0.75	8.28
Wu C.X. 2021 [58]	0.616	0.005	0.606	0.626			2.88	8.39
Wu H.P. 2009 [59]	0.714	0.0113	0.692	0.736			0.56	8.23
Xie 2021 [60]	0.675	0.0076	0.66	0.69			1.25	8.33
Zhang 2019 [63]	0.675	0.0059	0.663	0.687			2.07	8.37
Zhao 2013 [64]	0.692	0.0012	0.69	0.694			49.98	8.42
Total (fixed effects)	0.748	0.0008	0.747	0.75	882.121	<0.001	100	100
Total (random effects)	0.701	0.0211	0.66	0.742	33.266	<0.001	100	100

**Table 6:** Discriminatory ability of IL-6 clearance according to area under receiver operating Characteristics.

Study	ROC Area	Standard Error	Lower Limit of 95% CI	Upper Limit of 95% CI	Z	*p*-Value	Weight (%)—Fixed	Weight (%)—Random
Miguel-Bayarri 2012 [46]	0.86	0.04	0.782	0.938			76.42	67.92
Takahashi 2016 [53]	0.76	0.072	0.619	0.901			23.58	32.08
Total (fixed effects)	0.836	0.035	0.768	0.905	23.921	<0.001	100	100
Total (random effects)	0.828	0.0467	0.736	0.919	17.737	<0.001	100	100

## Data Availability

The original contributions presented in the study are included in the article. Further inquiries can be directed to the corresponding author.

## Supplementary Materials

The following supporting information can be downloaded at https://www.mdpi.com/article/10.3390/biom15030407/s1: Table S1: Excluded studies and reasons for exclusion; Table S2: QUIPS domains for risk of bias assessment; Table S3: Risk of bias ratings; Table S4: Full dataset used for meta-analysis.
