# Supplementary material for: IL-6 Baseline Values and Dynamic Changes in Predicting Sepsis Mortality: A Systematic Review and Meta-Analysis
Source: Biomolecules. 2025 Mar 13;15(3):407. doi: 10.3390/biom15030407 (PMC11940105; doi:10.3390/biom15030407)
Supplement: Supplementary file 1 [file biomolecules-15-00407-s001.zip › biomolecules-3457054-supplementary.pdf]

Supplementary Materials

The supplemental material consists of 4 tables:

Table S1. Excluded Studies and Reason for Exclusion.

Study ID	Title	Reason for exclusion
Amancio 2013 [17]	The Innate Immune Response in HIV/AIDS Septic Shock Patients: A Comparative Study	WRONG POPULATION
Viallon 2008 [18]	Can emergency physicians identify a high mortality subgroup of patients with sepsis: role of procalcitonin	INSUFFICIENT DATA ON PROGNOSTIC FACTOR
Calandra 1991 [19]	High circulating levels of interleukin-6 in patients with septic shock: evolution during sepsis, prognostic value, and interplay with other cytokines. The Swiss-Dutch J5 Immunoglobulin Study Group4	WRONG POPULATION
Kandaswamy 2018 [20]	Comparative Evaluation of Procalcitonin and Interleukin-6 as Diagnostic and Prognostic Biomarkers for Sepsis	WRONG POPULATION
Kellum 2007 [21]	Understanding the inflammatory cytokine response in pneumonia and sepsis: results of the Genetic and Inflammatory Markers of Sepsis (GenIMS) Study	WRONG OUTCOME
Tschaikowsky 2011 [22]	Predictive value of procalcitonin, interleukin-6, and C-reactive protein for survival in postoperative patients with severe sepsis	INSUFFICIENT DATA ON PROGNOSTIC FACTOR
Wunder 2004 [23]	Are IL-6, IL-10 and PCT plasma concentrations reliable for outcome prediction in severe sepsis? A comparison with APACHE III and SAPS II	INSUFFICIENT DATA ON PROGNOSTIC FACTOR
Jahn 2020 [24]	Score performance of SAPS 2 and SAPS 3 in combination with biomarkers IL-6, PCT or CRP	WRONG PROGNOSTIC FACTOR
Ebihara 2021 [25]	Cytokine Elevation in Severe COVID-19 From Longitudinal Proteomics Analysis: Comparison With Sepsis	WRONG STUDY DESIGN, OUTCOME
Jekarl 2019 [26]	Diagnosis and Prognosis of Sepsis Based on Use of Cytokines, Chemokines, and Growth Factors	PROGNOSTIC FACTOR NOT INCLUDED IN OUTCOME STUDY
Davoudian 2022 [27]	A cytokine/PTX3 prognostic index as a predictor of mortality in sepsis	WRONG PROGNOSTIC FACTOR, WRONG OUTCOME
Lorente 2016 [28]	Association between Interleukin-6 Promoter Polymorphism (-174 G/C), Serum Interleukin-6 Levels and Mortality in Severe Septic Patients	WRONG STUDY DESIGN, WRONG OUTCOME
Frimpong 2022 [29]	Cytokines as Potential Biomarkers for Differential Diagnosis of Sepsis and Other Non-Septic Disease Conditions	WRONG POPULATION
Gentile 2014 [30]	Is There Value in Plasma Cytokine Measurements in Patients with Severe Trauma and Sepsis?	NOT COHORT STUDY
Harbarth 2001 [31]	Diagnostic value of procalcitonin, interleukin-6, and interleukin-8 in critically ill patients admitted with suspected sepsis	WRONG OUTCOME
Jekarl 2015 [32]	Diagnosis and evaluation of severity of sepsis via the use of biomarkers and profiles of 13 cytokines: a multiplex analysis	WRONG OUTCOME
Oda 2005 [33]	Sequential measurement of IL-6 blood levels in patients with systemic inflammatory response syndrome (SIRS)/sepsis	WRONG STUDY DESIGN

Table S2. QUIPS Domains for Risk of Bias Assessment.

Domain	Criteria	Risk of bias rating
1. Study enrolment	The study population and key characteristics are thoroughly described. Eligibility criteria and recruitment processes are transparently detailed. The study period and location are specified. Adequate participation of eligible individuals is ensured, with no exclusions based on biomarker values or patient outcomes.	a. Low Risk: All criteria are met, suggesting minimal differences between participants and eligible non-participants.<br>b. Moderate Risk: Some criteria are unclear or problematic.<br>c. High Risk: Multiple criteria are problematic, indicating potential differences between participants and eligible non-participants.
2. Study attrition	No unwarranted exclusions or significant non-participation are observed. Efforts to collect data from participants who dropped out are described (if applicable). No major differences exist between participants who completed the study and those who did not (in cases of justified exclusions).	a. Low Risk: All criteria are met, suggesting minimal differences between completing and non-completing participants.<br>b. Moderate Risk: Some criteria are unclear or problematic.

		c. High Risk: Multiple criteria are problematic, indicating potential differences between completing and non-completing participants.
3. Biomarker measurement	The laboratory technique or method for measuring the biomarker is clearly reported. The same method and setting are used for all participants. Measurement methods are accurate, valid, consistent, and reliable. Continuous variables are handled appropriately, with rationale provided for any categorization. An adequate proportion of the study sample has complete data, or appropriate imputation methods are used.	a. Low Risk: All criteria are met, suggesting minimal impact on the relationship between the biomarker and outcome.<br>b. Moderate Risk: Some criteria are unclear or problematic.<br>c. High Risk: Multiple criteria are problematic, indicating potential impact on the relationship between the biomarker and outcome.
4. Outcome measurement	Mortality assessment methods are accurate, valid, consistent, and reliable. The method and setting of measurement are the same for all participants.	a. Low Risk: All criteria are met, suggesting minimal bias in outcome measurement.<br>b. Moderate Risk: Some criteria are unclear or problematic.<br>c. High Risk: Multiple criteria are problematic, indicating potential bias in outcome measurement.
5. Adjustment for other prognostic factors	All the important confounders (age, severity score) are measured and clearly defined. The method and setting of measurement are the same for all participants. Validated scales are used for measurement. The two potential confounders are accounted for in the study design or analysis. Appropriate methods are used to handle missing data.	a. Low Risk: All criteria are met, suggesting minimal confounding effects.<br>b. Moderate Risk: Some criteria are unclear or problematic.<br>c. High Risk: Multiple criteria are problematic, indicating potential confounding effects.
6. Statistical analysis and reporting	Sufficient data are presented to assess the adequacy of the analysis. The model-building strategy is acceptable and based on a conceptual framework. The analysis is appropriate for the study design. There is no selective reporting of results.	a. Low Risk: All criteria are met, suggesting minimal bias in analysis and reporting.<br>b. Moderate Risk: Some criteria are unclear or problematic.<br>c. High Risk: Multiple criteria are problematic, indicating potential bias in analysis and reporting.

Table S3. Risk of Bias Ratings.

Study ID	D1-Study participation	D2-Study attrition	D3-Prognostic factor measurement	D4-Outcome measurement	D5-Study confounding	D6-Statistical analysis and reporting
Andaluz-Ojeda 2012	Low	Moderate	Moderate	Moderate	Moderate	High
Belli 2022	Low	Moderate	Moderate	Moderate	High	High
Beneyto 2016	Low	Moderate	Low	High	Low	Moderate
Eidt 2016	Moderate	Moderate	Moderate	Moderate	High	High
Frencken 2017	Low	Low	Low	Moderate	Low	Moderate
Jekarl 2013	Low	High	Moderate	Moderate	High	High
Jiang 2019	Low	Moderate	Low	Moderate	Moderate	High
Karamouzos 2021	Moderate	Low	Low	Moderate	Moderate	High
Karampela 2022	High	Moderate	Low	Moderate	Moderate	High
Liu S 2021	Moderate	Low	Low	Moderate	Low	Low
Liu J 2021	Low	Low	Low	Low	Low	Low
Matsumoto 2018	Moderate	Moderate	Low	Low	Moderate	Low
Miguel-Bayarri 2012	Low	Low	Low	Low	Low	Low
Oberholzer 2005	High	Moderate	Low	Low	Low	High
Phua 2008	Low	Moderate	Moderate	Moderate	High	High
Ricarte-Bratti 2017	Moderate	Moderate	Moderate	Moderate	High	High
Rios-Toro 2017	Low	Low	Moderate	Low	Low	Moderate
Siddiqui 2019	Moderate	Moderate	High	Moderate	Low	Moderate
Song 2019	Moderate	Moderate	Low	Moderate	High	Moderate
Takahashi 2016	Moderate	Moderate	Moderate	Moderate	Low	High
Thao 2018	Moderate	High	High	Moderate	Moderate	High
Turan 2023	Low	Low	Moderate	Moderate	Low	Low
Vivas 2021	Low	Moderate	Moderate	Moderate	Moderate	High
Weidhase 2019	High	Moderate	High	Moderate	High	High
Wu CX 2021	Moderate	Low	Low	Moderate	Moderate	Low
Wu HP 2009	Low	Moderate	Low	Moderate	Moderate	Moderate
Xie 2021	Low	Low	Moderate	Moderate	High	High
Xie 2023	Low	Moderate	Low	Moderate	High	Moderate
Yu 2022	Low	Low	Low	Low	Low	Low
Zhang 2019	Moderate	Moderate	Moderate	Moderate	Low	Low
Zhao 2013	Low	Low	Low	Low	Moderate	Low

Table S4. Full Dataset Used for Meta-Analysis.

Study ID	SOFA	APACHE-II	BASELINE IL-6 (pg/mL)	IL-6 clearance (pg/mL)	IL-6 Logistic regression (95% CI)	AUROC to determine outcome (95% CI)
Andaluz-Ojeda 2012	7.7 in S vs 8.7 in NS; mean	19.5 in S vs 25 in NS; mean	97.2 (290.9) in S vs 637.9 (12666.0) in NS; median (IQR)	NR	Adjusted HR at D3: 1.86 (1.08–3.20); D28: 2.00(1.22 - 3.27)	NR
Belli 2022	NR	NR	446 (49-2540) in S vs 1198 (180-8646) in NS; median (IQR)	NR	HR= 1.000 (1.000–1.000)	NR
Beneyto 2016	6.6 (2.9) in S vs 9.1 (3.2) in NS; mean(SD)	14.6 (5.8) in S vs 20.2 (5.6) in NS; mean(SD)	Log IL-6= 4.8 (1.4) in S vs 5.6 (1.4) in NS; mean(SD)	Log transformed IL-6 D3= 3.6 (1.2) in S vs 5.6 (1.4) in NS; D7= 3.3 (1.2) in S vs 4.8 (1.2) in NS; mean(SD)	Admission: Log IL-6 OR=1.62(1.24-2.13); Day3: Log IL-6 OR= 2.69 (1.64–4.40)	ROC curves on admission and D3, AUROC not reported
Eidt 2016	7.2(3.0) in severe sepsis group vs 7.6(5.2) in septic shock group; mean (SD)	11.4(4.7) in severe sepsis vs 16.0(6.7) in septic shock; mean (SD)	28.4 (11.7–88.3) in S vs 94.2 (25.4–202.5) in NS; mean(SD)	NR	NR	Baseline: 0.669 ± 0.080
Frencken 2017	NR	NR	Log IL-6= 7.6 (5.3-8.8) in early deaths group (D4) vs 6.0 (4.1-7.7) in intermediate deaths (D28)	Log transformed IL-6 in D2= 6.6 (4.9-8.4) in early deaths group(D4) vs 4.8 (3.5-6.0) in intermediate deaths group (D28)	Admission: RR=1.13 (0.91–1.41); D4: RR= 1.03 (0.86–1.23)	NR
Jekarl 2013	NR	NR	S: 305.2 pg/mL; NS: 1018.8 pg/mL; mean	IL-6 concentrations measured at 12h, 24h, 48h, 72h (details represented on graph)	NR	NR;
Jiang 2019	7(4-13) in S vs 10(6-15) in NS; median(25th-75th)	NR	34.2(15.7-55.5) in S vs 124.3(49.3-171.9) in NS; median(IQR)	D3: 23.3 (8.6–45.3) in S vs 76.0 (29.0–153.6) in NS; D7: 21.9 (11.7–89.0) in S vs 44.9 (10.2–107.2) in NS; median (IQR)	OR= 1.033 (1.007–1.061)	Baseline: 0.757
Karamouzos 2021	6.5(4) in S vs 9.2(3.6) in NS; mean(SD)	19(6.6) in S vs 22(6.3) in NS; mean(SD)	9 (4-24) in S vs 22(10-52) in NS; median(IQR)	NR	OR= 1.002 (0.996 - 1.008)	NR
Karampela 2022	10(3.3); mean(SD)	23(7.2); mean(SD)	16.5(6-385) in sepsis group vs 74.4(10-444) in septic shock group; median (IQR)	D7: 25 (4.6–419) in sepsis group vs 20.5 (6–487) in septic shock group	HR= 1.70 (1.05–2.74)	NR
Liu S 2021	8(6-11); median(25th-75th)	20.79(6.89); mean(SD)	75.88(10.04) in S vs 113.77 (11.69) in NS; mean(SD)	NR	OR= 1.017(1.005–1.028)	0.849 (0.799–0.890)
Liu J 2021	6.2 (3.6) in S vs 9.9 (4.6) in NS; mean(SD)	25.1 (8.8) in S vs 36.5 (7.6) in NS; mean(SD)	217.6 (103.3–962.4) in S vs 4809.0 (247.2–5000.0) in NS; median(IQR)	NR	OR= 1.001 (1.000–1.001)	0.785 (0.647–0.923)
Matsumoto 2018	9.0 (5.0–11.0); median(IQR)	21.0 (16.0–29.0); median (IQR)	Log IL-6 at D1: 3.5 (2.1-4.7); median(IQR)	Log IL-6 D2: 2.8 (1.9-3.5); D4:2.4(1.5-3.2); D6:2(1.2-2.8); median(IQR)	The maximum values from three days (D1, D2, D4): 19.62(3.47-110.80)	D1: 0.899
Miguel-Bayarri 2012	8(13-2) in S vs 10(16-3) in NS; median(25th-75th)	16(29-2) in S vs 25(30-9) in NS; median(IQR)	Log Il-6= 4.7(8.3-2) in S; 6.1(9.9-3.3) in NS; median (25th-75th)	D3: 3.6(6.1-0.47) in S, 5.8(9.9-3.1) in NS; D7: 3.3(5.8-1.5) in S, 4.9(6.7-2.6) in NS; median (IQR)	Admission: Log IL-6 OR= 1.98(1.27-3.09); D3: Log IL-6 OR= 2.6(1,43-4.71)	Admission: 0.74 (0.63-0.86); D3: 0.86 (0.78-0.94); D7: 0.80 (0.67-0.93)
Oberholzer 2005	NR	21.6(8.2); mean(SD)	Log IL-6 = 2.78 (0.86) in S vs Log IL-6 = 3.66 (1.08) in NS; mean(SD)	Plasma IL-6 concentrations determined on D1 through D4 (details in graph)	Not significant (p value)	NR
Phua 2008	10.1(3.0) in S vs 12.7(4.4) in NS; mean(SD)	23.1(7.5) in S vs 32.3(8.7) in NS; mean(SD)	NR	NR	NR	Admission: 0.77(0.65-0.89)
Ricarte-Bratti 2017	5.5 (2) in S vs 10.8(3.4) in NS; mean(SD)	13.9(5.5) in S vs 22.9(8.6) in NS; mean(SD)	161(24) in S vs 203 (26) in NS; mean(SD)	After 72h: 121(17) in S vs 205(26) in NS; mean(SD).	NR	NR

Rios-Toro 2017	7(1-16); median (IQR)	19(6-43); median (IQR)	62 (12–156) in S vs 44 (10–92) in NS; median (25th-75th)	D0 to D2: -28 (-73; +18) % in S vs -2 (-50; +92) % in NS; D0 to D5: -86 (-98; -44) % in S vs +4 (-81; +116) % in NS	NR	50% decrease in serum IL-6 from D0 to D5: AUC=0.706
Siddiqui 2019	NR	NR	51.8 (13.1-198.7); 43.2 (13.9-98.5); 29.4 (13.9-59.9); median(IQR)	NR	HR = 1.46 (1.11 - 1.92)	NR
Song 2019	8(4-11); median(25th-75th)	21(13-30); median(25th-75th)	89.9 (45.2–272.6) in sepsis group; 1378.6 (256.4–11,062.1) in septic shock group; median (25th-75th)	In septic shock group follow-up IL-6 within 24h of discharge (recovery or death): 21.5(10.2–51.7) in S vs 9976.5 (4651.2–71,048.3) in NS	HR= 1.001 (1.000–1.002)	0.674
Takahashi 2016	11(8-15); median(25th-75th)	28(21-36); median(25th-75th)	720 (183-7656); median (25-75)	D3: 116(35-406); D5: 78(27-204); D7: 51(31-118); median(25th-75th)	NR	D1: 0.654(0.514-0.795); D3: 0.760(0.616-0.904); D5: 0.813(0.687-0.940); D7: 0.883(0.788-0.978)
Thao 2018	9.1(2.9) in S vs 11.6(3.6) in NS; mean(SD)	18.0(5.8) in S vs 26.6(7.9) in NS; mean(SD)	413.3 in S; 530.0 in NS; mean	After 24h: 65.4 in S; 286.9 in NS; IL-6 rate of change in 24h: +0.8 (S) vs -0.7 (NS)	IL-6 clearance in 24h: ≥86% OR= 5.67(1.27–25.3); Il-6 clearance between 85% and 50% OR= 1.86 (0.44–7.94)	NR
Turan 2023	7.3 (3.6) in S vs 12.9(3.6) in NS; mean(SD)	17.8(8.3) in S vs 25.5(9.5) in NS; mean(SD)	76 (44–250) in S vs 108 (42–1137) in NS; median(IQR)	NR	NR	Baseline IL-6: 0.573(0.424-0.722)
Vivas 2021	NR	NR	68(7-300) in S vs 125(14-250) in NS; median(25th-75th)	48h after admission: 7(7-63.25) in S vs 14(7-81.25) in NS; median (25th-75th)	NR	NR
Weidhase 2019	7.6 (3.9) in S vs 10.1 (3.9) in NS; mean(SD)	26.6 (8.1) in S vs 28.0 (7.6) in NS; mean(SD)	600.6 (218.9 - 2743.5) in S vs 381.6 (170.3 - 1558.0) in NS; median(IQR)	48-72h: 114.2 [54.4;229.9] in S vs. 746.6 [261.8;1808.8] in NS; median(IQR)	NR	D1: 0.701; after 48-72h: 0.792
Wu CX 2021	7(4-11); median(25th-75th)	17.5(12.75-22); median(25th-75th)	NR	NR	OR= 1.66 (0.67 - 4.10)	0.616(0.512-0.720)
Wu HP 2009	NR	22.7(1.0) in S vs 28.7(2.3) in NS; mean(SD)	90.16(20.49) in S vs 294.31(79.18) in NS; mean(SD)	NR	OR= 1.00(0.99 - 1.01)	D1: 0.714 (0.538-0.891)
Xie 2021	6.5 (3.7) in S vs 9.7 (4.8) in NS; mean(SD)	NR	205.30 (11.50–5000.00) in S vs 3499.00 (13.53–5000.00) in NS; median(25th-75th)	NR	OR=1.000(1.000–1.001)	0.675 (0.534–0.816)
Xie 2023	5.6 (2.1) in S vs 8.8 (3.4) in NS; mean(SD)	NR	85.70 (2.10 to 5000.00) in S vs 242.80 (16.45 to 5000.00) in NS; median(25th-75th)	D3: 39.59 (0.32 to 622.60) in S vs 185.00 (2.32 to 5000.00) in NS; median (25th-75th)	D1: OR=1.000 (1.000 - 1.001); D3: 1.007 (1.003 - 1.010)	NR
Yu 2022	5(3-6); median(25th-75th)	33.56(11.91); mean(SD)	92.22(21.53–201.12) in sepsis group; median (IQR)	NR	OR= 0.999 (0.996-1.002)	NR
Zhang 2019	6(4-8) in sepsis group; 4(2-10) in septic shock group; median(25th-75th)	NR	74.187 (250.10–379190.90) in S vs 178.335 (260.32–449450.00) in NS; median(25th-75th)	NR	OR=1.02(1.00-1.00)	0.675 (0.534–0.816)
Zhao 013	NR	NR	18.9 (7.1-59.4) in S vs 61.2 (18.3-194.2) in NS; median(IQR)	NR	OR= 1.002 (1.000-1.004)	0.692 (0.641-0.744)
